# Effects of Pidotimod on recurrent respiratory infections in children with Down syndrome: a retrospective Italian study

**DOI:** 10.1186/s13052-020-0797-5

**Published:** 2020-03-13

**Authors:** Diletta Valentini, Chiara Di Camillo, Nadia Mirante, Valentina Marcellini, Rita Carsetti, Alberto Villani

**Affiliations:** 1 Pediatric and Infectious Disease Unit, Bambino Gesù Children’s Hospital, IRCCS, Piazza S. Onofrio 4, 00165 Rome, Italy; 2 Immunology Research Area, B-cell development Unit, Bambino Gesù Children’s Hospital, IRCCS, Rome, Italy; 3 Immunological Diagnosis Unit, Bambino Gesù Children’s Hospital, IRCCS, Rome, Italy

**Keywords:** Down syndrome, Pidotimod, Recurrent respiratory infections

## Abstract

**Background:**

Children with Down syndrome (DS) show a high susceptibility to recurrent infections (RI), caused by immune defects and abnormalities of the airways. Our goal was to investigate the effects of Pidotimod on RI prevention in children with DS, comparing immune and clinical parameters before (T0) and after (T1) the treatment with Pidotimod.

**Methods:**

The study was conducted at the Down syndrome outpatient Center of Bambino Gesù Children’s Hospital, in Rome. We reviewed the medical records of all children with a positive history for RI and who received oral prophylaxis of Pidotimod from September 2016 to February 2017.

**Results:**

Thirty-three children met the inclusion criteria (males: 51.5%; average age: 6 years ±SD: 3). We found a significant decrease in the number of children with upper respiratory infections (82% at T0 vs 24% at T1; *p* = 0,0001) and with lower respiratory infections (36% at T0 vs 9% at T1; *p* = 0.003) after treatment with Pidotimod. We also demonstrated a significant decrease in the number of children hospitalized for respiratory infections (18% at T0 vs 3% at T1; *p* = 0.03). We measured T and B cells in the peripheral blood and B cell function in vitro at T0 and T1. We found that the response to CpG improved at T1. A significant increase of B cell frequency (*p* = 0.0009), B cell proliferation (*p* = 0.0278) and IgM secretion (*p* = 0.0478) were observed in children with DS after treatment.

**Conclusions:**

Our results provided evidence that Pidotimod may be able to prevent RI in children with Down syndrome.

## Background

Down syndrome (DS) is the most common chromosomal abnormality among live-born infants. The number of people with DS living in the United States has grown from 49,923 in 1950 to 206,366 in 2010 [1]. There are no population-based registries in Italy, but the prevalence of people with DS living in Italy is estimated at approximately 30,000. More than half of them are over 25, and 3000 are over 45 years old [2].

Life expectancy in children with DS has increased significantly over the last decade, but children with DS remain at higher risk of neonatal and infant mortality than children without DS, respectively (1.65% vs. 0.36 and 4% vs. 0.48%) [3].

Important causes for increased mortality in DS are congenital heart disease, other congenital anomalies (e.g. nervous system, respiratory system, gastrointestinal tract, genito-urinary system and musculoskeletal system), leukemia, testicular cancer and sepsis. Furthermore, respiratory tract infection (RTI) represents the second leading cause of death, in children with DS, up to the age of 18 [4–8].

Congenital heart diseases, abnormalities of the airways, generalized hypotonia and swallowing dysfunction predispose children with DS to frequently contract and have more severe RTI [9].

Furthermore, patients with DS show multiple defects in both numbers and function of innate and adaptive immunity [9].

Recently, other authors and we reported that DS is associated with a primary defect of the B-cell compartment, characterized by a reduced number of all B-cells in the peripheral blood and especially of memory B cells. IgM memory B cells representing the first-line defense against infection [10] are 1/3 of normal, whereas highly specific switched memory B cells are 7–10 fold less than expected [11, 12]. We have also shown that children with DS respond poorly to primary immunization and their protection may require tailored vaccination protocols [13].

As a consequence of these defects, children with DS, usually, show a high susceptibility to recurrent infections (RI), characterized by increased severity and a prolonged course [14].

In these patients, the development of prevention strategies of RI is necessary. Currently, the American Academy of Pediatrics recommends pneumococcal and influenza vaccine and the use of palivizumab in children with DS, who are at risk of severe respiratory syncytial virus (RSV) infections (eg, congenital heart disease, airway clearance issues, prematurity) [15, 16].

In addition to vaccination and monoclonal antibodies, immunostimulants are widely employed in the common practice to prevent RI in susceptible individuals, but there are no recommendations in children with DS.

Pidotimod (3-L-py- roglutamyl-L-thiaziolidine-4carboxylic acid) is a synthetic dipeptide molecule exerting immunomodulatory activities [17]. Pidotimod is a highly purified molecule, which is quickly absorbed by the digestive tract, the bioavailability is 45% not modified by food intake, and is excreted un-metabolized through renal pathway [18].

In our study, we investigated retrospectively, the effects of Pidotimod on RI prevention in children with DS, comparing immune and clinical parameters before and after the treatment with Pidotimod.

## Materials and methods

The retrospective study was conducted at the Down Syndrome outpatient Center of Bambino Gesù Children’s Hospital, in Rome. We reviewed, retrospectively, the medical records of all children with a positive history for RI and who received oral prophylaxis with a 400 mg dose of Pidotimod a day for the first twenty days of each month for 6 months (from September 2016 to February 2017).

Signs and symptoms to define RI for lower respiratory infections: two or more episodes/year; for upper respiratory infections: six or more episodes/year; for urinary tract infections: three or more episodes/year and for gastroenteritis: three or more episodes/year [19–21].

During the annual visit, the medical doctor routinely collected medical information about the number, the type of infections and frequency admissions for infections of patients in a year. As recommended by Academy of Pediatrics guidelines, the physician annually ordered to children with DS the blood draw for celiac disease, complete blood count, ferritin, C-reactive protein and thyroid tests [16]. The immunological tests carried out in the children affected by RI in order to identify defects of the immune system, as the good clinical practices.

We created an anonymous database, in which we reported the following information: age, gender, patients schooled or not, siblings schooled or not, smokers who lived with the children or not, flu immunization, the frequency and type of infections and related hospitalizations. In the database, we also reported T and B cells in the peripheral blood and B cell function in vitro*.*

We compared the clinical and immunological parameters for each patient in the period from September 2015 to February 2016 (T0: before Pidotimod) and the period from September 2016 to February 2017 (T1: after Pidotimod).

The local institutional review board approved the study protocol and waived the need for informed consent owing to the retrospective nature of the study.

### Cell isolation and flow cytometry analysis

Heparinized PBMCs were isolated by Ficoll PaqueTM Plus (Amersham Pharmacia Biotech) density-gradient centrifugation, counted and stained with the appropriate combination of fluorescent labeled antibodies and analyzed by flow cytometry [22]. Dead cells were excluded from analysis by side/forward scatter gating. All analyses were performed on a LSRFortessa-X20 (BD Biosciences) interfaced to PC FACSDiva software. A total of 500,000 events per sample were analyzed.

### B-cell proliferation and immunoglobulin production in vitro

Before stimulation, peripheral blood lymphocytes were labeled with CMFDA (5-Chloromethylfluorescein diacetate) at a final concentration of 0.1 mg/mL (CellTrace CFSE; Thermo Fisher Scientifics,). The cells were cultured at 5 × 10^5^ cells per well in 96-well plates (Becton Dickinson, San Jose, CA, USA) in complete RPMI 1640 (InvivoGen, San Diego, CA, USA), supplemented with 10% FBS (Hyclone Laboratories, Logan, UT, USA), 2% L-glutamine (Gibco BRL), 5 × 10–5 M 2-beta-mercaptoethanol (Sigma-Aldrich, St. Louis, MO, USA), and 20 mg/mL gentamycin (Gibco BRL). CpG ODN (Hycult Biotechnology, The Netherlands) was added at the concentration of 2.5 mg/mL [23].

### Elisa

The amount of IgM, IgG, and IgA present in the culture medium after seven days of stimulation was measured using an in-house ELISA. Plates were coated with anti-IgM/IgG/IgA antibodies (Jackson Immunoresearch, Cat #109–006-064). Peroxidase-conjugated anti-IgM (cat #109–36-129), anti-IgG (cat #109–036-008), or anti-IgA (cat #109–036-011) (all from Jackson Immunoresearch) were used as secondary antibodies. Purified serum IgG and IgA, and IgM purified from Myeloma cell lines (Jackson Immunoresearch) were used as standards.

### Statistical analysis

Categorical data were reported as numbers, percentages, and continuous variables as mean and ± SD. We carried out a descriptive analysis for all parameters. McNemar tests for nominative variables were performed to compare the clinical results before and after Pidotimod (T0 vs T1). The unpaired Student’s t-test was applied to the analysis of statistical difference. A level of *p* < 0.05 was considered statistically significant. Statistical analysis were computed with STATA 12 for Windows.

## Results

### Study population

In Table 1, we report the characteristics of the study population. Thirty-three children met the inclusion criteria (males: 51.5%; average age: 6 years ±SD: 3), almost all attended school (94%), 23 children (70%) had at least one sibling in school, 13 subjects (40%) lived with a smoker. We had information about flu immunization for 27 subjects (27/33; 82%): 14 of whom received flu vaccination (52%).

**Table 1:** Characteristics of the study population (*N* = 33)

	N	%
**Age**, years *[mean, SD]*	5.7	2.9
**Gender,** male	17	51.5
**Attending School**	31	93.9
**Attending school siblings**	23	69.7
**Parental smokers**	13	39.4
**Influenza immunization**^**a**^	14	51.9

^a^ We found this information only for 27 patients, thus the

percentage was calculated on 27 subjects (14/27, 51,9%)

### Clinical results

**Fig. 1:**
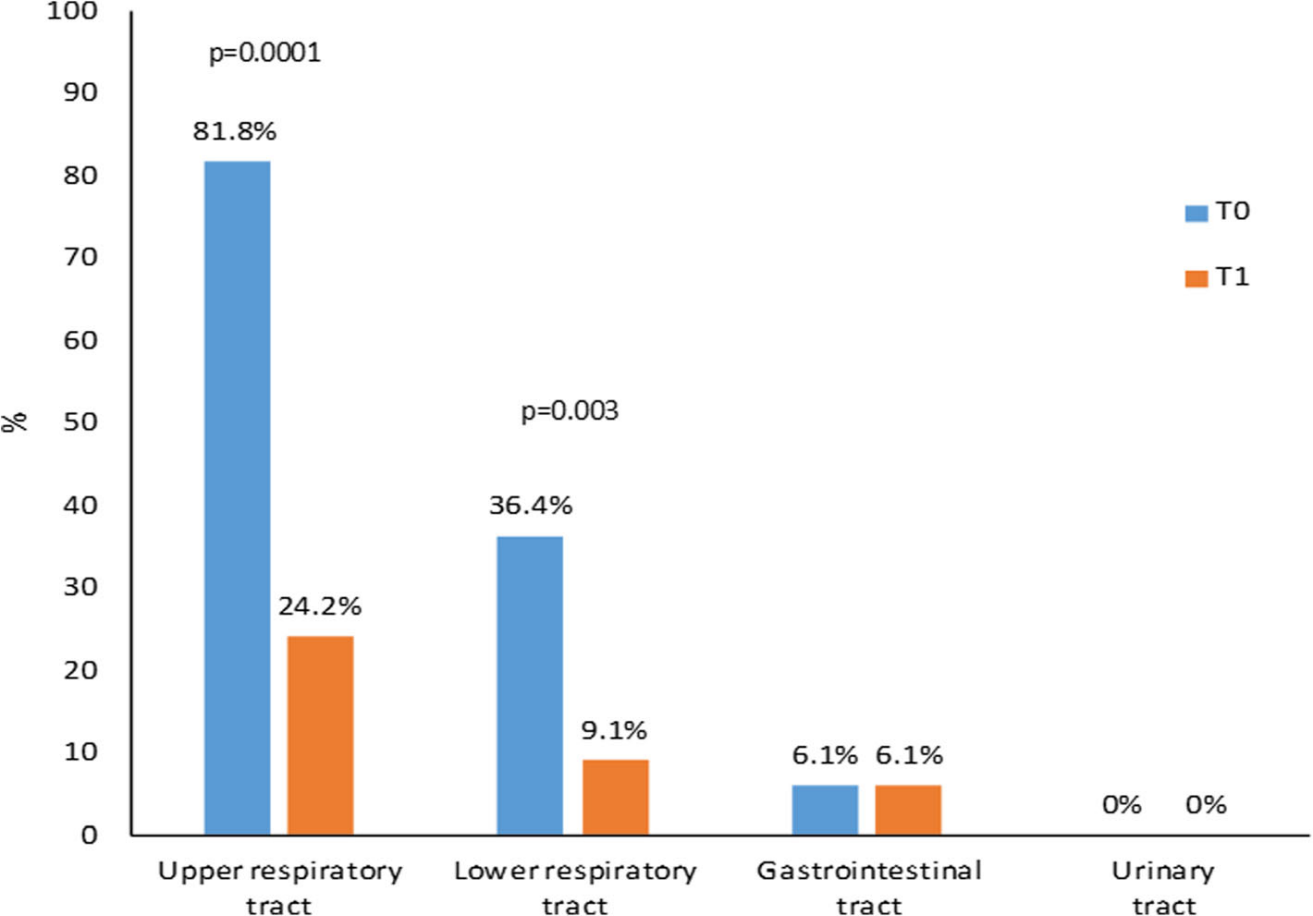
Percentage of children with RI by type at T0 and at T1 (*N* = 33)

As shown in Fig. 1, there was a significant lower number of children with upper respiratory infections at T1 compared to T0 (27/33, 82% at T0 vs 8/33, 24% at T1; *p* = 0,0001). We also found a significant decrease in the number of children with lower respiratory infections at T1 compared to T0 (12/33, 36% at T0 vs 3/33, 9% at T1; *p* = 0.003). However, in children (*n* = 2) with gastrointestinal infections no significant differences were shown (*p* = 1). While no one referred RI to urinary tract. More than one RI could affect each patient.

**Fig. 2:**
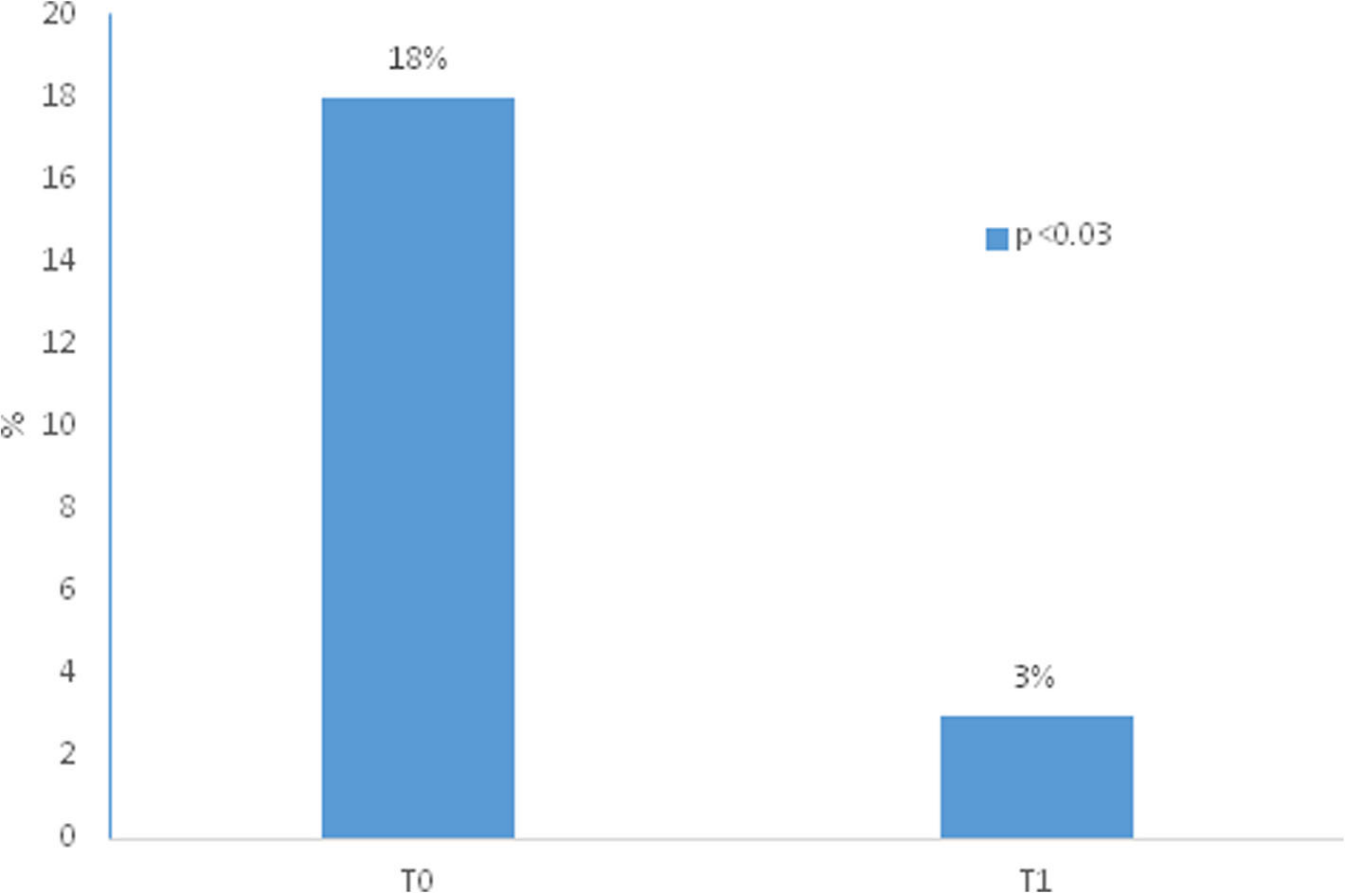
Proportions of patients hospitalized at T0 and at T1 (*N* = 33)

We found, as reported in Fig. 2, a significant decrease in the number of children hospitalized for respiratory infections at T1 compared to T0 (6/33, 18% at T0 vs 1/33, 3% at T1; *p* = 0.03).

### Immunological results

In order to investigate whether Pidotimod treatment has measurable effects on the immune system, we compared T and B cells in the peripheral blood and B cell function in vitro at T0 and T1.

We were unable to demonstrate any effect of the treatment with Pidotimod on either B or T cells in the peripheral blood. Lymphocyte population frequencies and absolute numbers remained unchanged (Table 2).

**Table 2:** B and T immunofenotype at T0 and T1

	T0		T1		***P*** values
	Mean	±SD	Mean	±SD	
**Lymph**	2041.2	974.4	2092.7	842.9	0.677
**B cells**	222.7	135.8	237.4	142.6	0.500
**Memory B**	23.9	14.6	28.0	20.7	0.161
**Mature**	156.4	106.4	161.7	112.5	0.811
**Transitional**	40.3	39.2	44.3	35.0	0.460
**Plasma cells**	12.7	11.9	15.4	12.9	0.317
**T cells**	1766.2	1481.3	1495.4	678.6	0.294
**CD4**	657.1	368.0	632.1	298.6	0.628
**CD8**	774.0	579.3	729.9	437.4	0.471
**NK cells**	273.3	168.5	302.3	200.4	0.379
**CD4-RO**	296.1	134.3	305.4	126.6	0.710
**CD4-RA**	352.4	299.9	320.2	224.1	0.360
**CD8-RO**	264.3	244.7	228.2	129.1	0.302
**CD8-RA**	506.7	385.9	491.4	338.2	0.746

The function of B cells can be measured in vitro by the stimulation with the TLR9 ligand CpG .

**Fig. 3:**
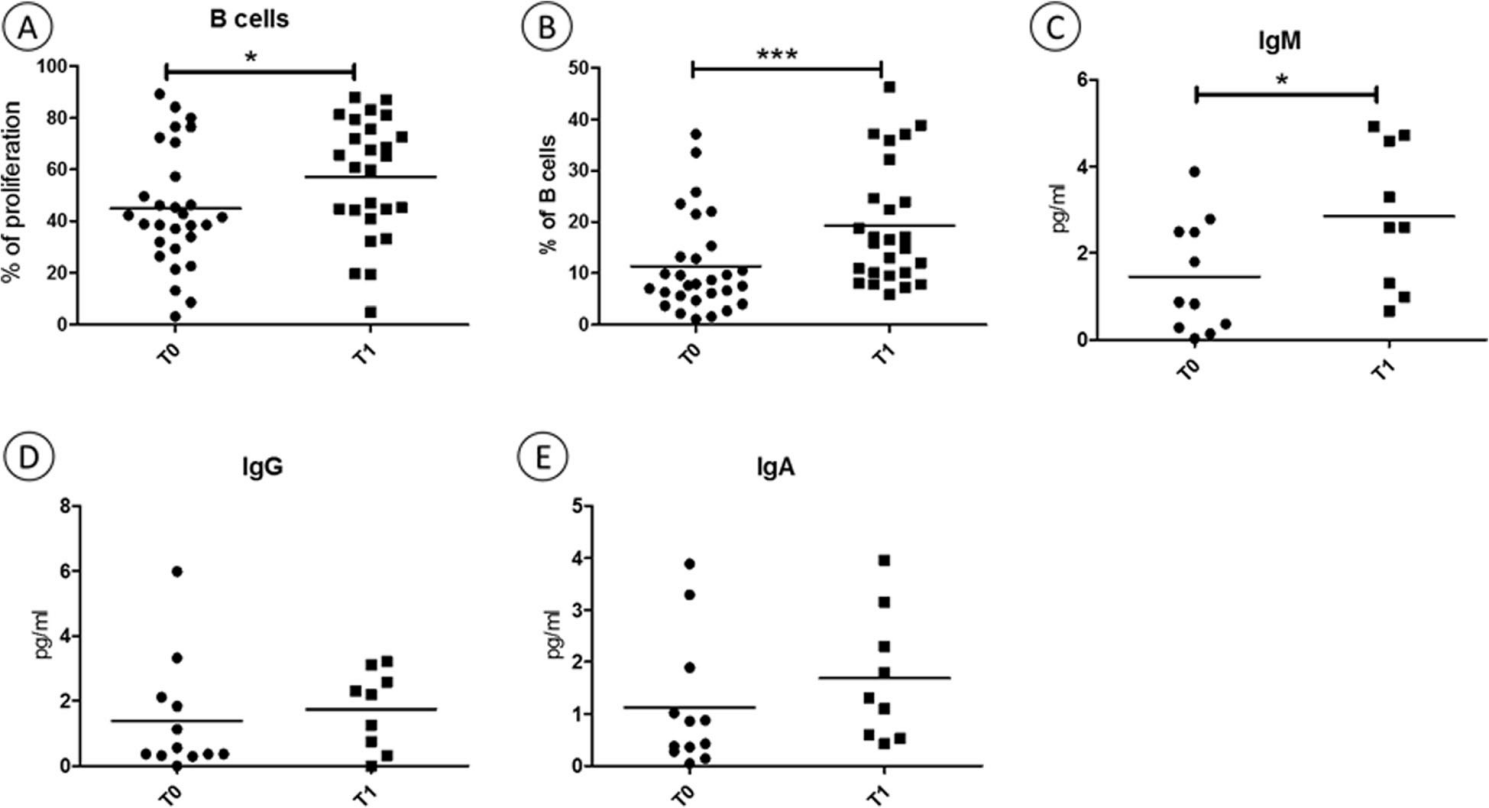
The function of B cells measured in vitro by the stimulation with the TLR9 ligand CpG

Upon exposure to CpG mature naïve B cells increase their survival and memory B cells proliferate and produce antibodies. We found that the response to CpG improved at T1. A significant increase of B cells frequency (*p* = 0.0009), B cell proliferation (*p* = 0.0278) and IgM secretion (*p* = 0.0478) were observed in children with DS after treatment (Fig. 3).

## Discussion

Many children with DS have frequent RTI, and these are often more severe and prolonged than similar infections in their peers [24].

Children with DS have a higher incidence of hospitalizations, often associated with longer and more complicated stays as well as with a higher incidence of acute lung injury and acute respiratory distress syndrome [25, 26].

It is, therefore, important to identify effective interventions to prevent and treat these infections. Unfortunately, the studies proving the management of RTI in children with DS are few and far between, the evidence base requires further investigations in these populations [27].

Children with DS should be vaccinated according to the national schedule for immunization, including both obligatory and recommended vaccines. The Advisory Committee on Immunization Practices has also recommended the sequential administration of the 13-valent pneumococcal conjugate vaccine followed by the 23-valent pneumococcal polysaccharide vaccine in children over 2 years of age who are at a higher risk of pneumococcal disease, such as those with chronic underlying diseases or who are immunocompromised [28]. Notwithstanding the numerous reports demonstrating that immune deficiency is a consistent feature of the syndrome, [14] children with DS are not included in the list of patients at higher risk for infection. We suggest that pneumococcal and influenza vaccines should be strongly considered in children with DS [13]. In addition, Palivizumab for RSV prophylaxis is indicated for DS children with congenital heart disease or prematurity.

Prevention of RI is an ambitious goal. Mechanism-oriented studies alongside clinical intervention trials to test biologically plausible prevention ideas are necessary. In this scenario, administration of Pidotimod, a synthetic immunostimulant that modulates both the adaptive and the innate immune responses, could represent an innovative strategy.

Even if the mechanisms associated with these effects had not been investigated, the efficacy of the immunomodulator Pidotimod in reducing the rates of RTI in children with DS, was previously reported [29].

Recently, Zuccotti et al. found that co-administering the flu vaccine and Pidotimod boosted the production of antibodies against influenza [17].

In our study, we demonstrated a significant reduction of the number of children with RTI after treatment with Pidotimod, with a significant decrease in the percentage of hospitalizations for infectious diseases. These results confirm previous studies conducted in order to investigate the efficacy of Pidotimod to prevent RI both in children with DS and in pediatric population, as reported by Licari et al. and Niu et al. [29–31]

The preventive effect may depend on the activity of Pidotimod on immune cells. We found that Pidotimod treatment did not influence the number of B, T and NK cells in the peripheral blood, but improved the survival and function of B cells in vitro. Ex vivo stimulation with CpG measures the ability of B cells to react to TLR9 [24]. In response to CpG, memory B cells proliferate and differentiate into antibody producing cells. Whereas switched memory B cells produce IgG and IgA, IgM memory B cells secrete IgM. We showed that, after Pitodimod treatment, B cells increased in numbers, proliferated more and secreted higher amounts of IgM antibodies, whereas the concentration of IgG and IgA remained stable. Thus, the function of IgM memory B cell is significantly potentiated after Pitodimod treatment. The increased activity of IgM memory B cells in vitro may reflect an improved activity in vivo resulting in a more effective first-line protection and reduction of infection episodes in vivo. Further studies are necessary to evaluate whether Pidotimod may improve the response of other cell types to TLR ligands and re-enforce innate immune defenses, thus explaining the clinical effects of the treatment.

Pidotimod was well tolerated and we did not report any adverse events in our population.

The strength of our study was evidence of effectiveness of a preventive intervention, for RTI in children with DS, not only on clinical effects but also on immunological effects. That is especially evident on the survival and function of B cells in vitro. However, the retrospective data analysis and the lack of the control cases represented the limits of the present study. Further high-quality and large-scale randomized controlled trials are still required to provide confirmatory evidence.

## Conclusions

Our results provided evidence that Pidotimod may be able to prevent RTI in children with DS. Additional methodologically rigorous studies are necessary to guide clinicians on the identification of the best approaches to prevent and treat RI in children with DS.

## Data Availability

The datasets used and/or analysed during the current study are available from the corresponding author on reasonable request.

## Abbreviations

DS: Down syndrome
RI: Recurrent infections
RSV: Respiratory syncytial virus
RTI: Respiratory tract infection
